# Outcome assessment in veterinary pain studies: a pain in animals workshop (PAW) perspective

**DOI:** 10.3389/fpain.2025.1579155

**Published:** 2025-04-10

**Authors:** D. C. Brown, J. Coetzee, M. Gill, C. Johnson, D. P. Mohapatra, M. L. Oshinsky, S. A. Robertson, E. R. Smith, B. D. X. Lascelles

**Affiliations:** ^1^ Mars Veterinary Health, Vancouver, WA, United States; ^2^ Department of Anatomy and Physiology, Kansas State University, Manhattan, KS, United States; ^3^ National Institute of Neurological Disorders and Stroke/National Institutes of Health, Bethesda, MD, United States; ^4^ Center for Veterinary Medicine, Food and Drug Administration, Rockville, MD, United States; ^5^ Lap of Love Veterinary Hospice, Lutz, FL, United States; ^6^ Translational Research in Pain and Comparative Pain Research and Education Center, Department of Clinical Sciences, College of Veterinary Medicine, North Carolina State University, Raleigh, NC, United States; ^7^ Thurston Arthritis Center, UNC School of Medicine, Chapel Hill, NC, United States; ^8^ Center for Translational Pain Research, Department of Anesthesiology, Duke University, Durham, NC, United States

**Keywords:** pain, measurement, translational, behavior, therapeutic development

## Abstract

Biennially, the Pain in Animals Workshop (PAW) forum brings together scientists and clinicians to focus, across veterinary species and humans, on our shared passion of improving health through our ability to recognize and monitor pain. This collaboration has been instrumental in sharing current knowledge, identifying gaps, and aligning on the best paths forward in this challenging space. At the 2023 PAW held at the National Institutes of Health, Dr Dottie Brown delivered the inaugural Dr. Michele Sharkey PAW Lecture: “*Outcome Assessment in Veterinary Pain Studies: The Yellow Brick Road Continues*”. This perspectives article captures the content of that inaugural lecture and provides a reflection on how the PAW forums have been integral to the most recent wave of knowledge gain and awareness.

## Introduction

In 1996, Rimadyl® (carprofen caplets) was approved in the US as the first non-steroidal anti-inflammatory drug (NSAID) indicated for the relief of pain and inflammation associated with osteoarthritis in dogs. Effectiveness was evaluated using a veterinarian's assessment of pain and function, an owner's assessment of pain and function, and gait analysis. Nearly 30 years later, these three approaches continue to be the primary focus for the assessment of chronic osteoarthritis pain in dogs. While this may suggest stasis in the field of veterinary chronic pain outcome assessment, the last 15 years have seen marked advancements, with the Pain in Animal Workshops (PAW) being particularly pivotal in bringing awareness to and fostering innovation in this arena.

Following the approval of Rimadyl® for canine osteoarthritis pain, there was a quick succession of approvals of additional NSAIDs. There were educational campaigns on how to recognize chronic pain in dogs. Veterinary practice changed. The owner's mindset of “he's just slowing down because he's getting older” slowly shifted to proactively seeking care because “I think my dog has arthritis”. Not only did veterinary practice change, but research also changed. With proven effective therapeutics came the ability to perform robust studies evaluating the effectiveness of methods to diagnose and monitor osteoarthritis. Research shifted to a focus on capturing the owner's assessment of chronic pain in their dog.

The journey from concept, through validation, to acceptance of owner outcome assessment instruments for chronic pain research has often been uphill. This may be surprising, because veterinary clinical practice is foundationally driven by the owner's assessment of their pets—the presenting complaint. The continuation of the chosen treatment plan for the condition then revolves around the ability to reverse that owner's complaint. Although owner assessment has been, and continues to be, essential to monitoring treatment success in practice, the studies to establish the reliability and validity of owner assessments for research purposes were met with a series of objections as they underwent peer review.

## The objections

“Pain is subjective and subjective states cannot be measured.”

Twenty years ago, the veterinary medical research community was predominantly unaware of the body of science supporting the evaluation of the methodological quality and validation of reliable health outcome assessment instruments, or clinical metrology instruments (CMIs), to measure subjective health states. Concepts such as psychometric testing, construct validity, and internal consistency were not present in mainstream veterinary literature but had been borrowed from the human psychology space. This created a barrier to understanding instrument validation work from the veterinary medicine perspective. The historical requirement to have only veterinarians review manuscripts submitted to the mainstream veterinary journals, as well as the skepticism that revolved around a relative lack of references to veterinary publications, were barriers to the publication of those first articles describing the development of owner-completed CMIs in veterinary medicine.

“But our patients cannot speak.”

Eventually the veterinary field acquiesced to the concept that appropriate, established principles of questionnaire development can be used to reliably quantify subjective health states, including pain in animals. However, the next concern was that the field relied on self-report in humans, but animals cannot self-report. However, the inability to use self-assessment outcome measures is not exclusive to companion animal studies. Observer (relative or caregiver) completed assessments are commonly used in pediatric and cognitively impaired human populations. Although the subjective worlds of non-verbal subjects cannot be directly described by them, observable behaviors offer a basis for proxy assessments to be made by individuals knowledgeable about the subjects' behavior. The development of these tools is based on the concepts that an observer can evaluate the impact of pain via a global assessment using such things as facial characteristics, body posture, function, and movement patterns of the subject. Pain can interfere with activities of daily living, and a knowledgeable observer can reliably rate changes in behavior. While not initially intuitive, veterinary medicine now accepts that an owner's behavior-based assessment of chronic pain in their dog is similar to a caregiver's behavior-based assessment of pain in an adult who is nonverbal.

“But owners are not trained observers of pain.”

Once there was awareness that methodology existed for the development of reliable and valid CMIs, concerns were raised as to the owner's ability to be that observer. This concern stemmed from the approach to the measurement of acute pain (i.e., post-operative pain). The methods sections of papers describing interventions for acute pain detail the characteristics of the “trained observers” evaluating post-operative patients for pain and intervening according to a protocol: trained veterinary professionals have the greatest experience and expertise in understanding the behaviors of animals after surgery. However, with chronic pain, including conditions like osteoarthritis pain, cancer-associated pain, or oral and dental pain, the behaviors expressed that are indicative of pain are primarily occurring in their home environment. We now recognize that in the clinic environment, when animals are excited, stressed, or distracted, many of the behaviors indicative of chronic pain that the owners see at home cannot be evaluated reliably. The concept that the owner is indeed the most knowledgeable observer of their pet, which has been foundationally incorporated into clinical decision-making since the start of veterinary practice, is now also accepted as an informative component of clinical chronic pain research. As the initial hurdles to scientific acceptance of owner outcome assessment instruments for chronic pain were overcome, new questions started to emerge about how they fit into the clinical studies landscape.

## The new questions

The focus on owner assessment of chronic pain led to questions in the orthopedic research community that could sometimes spark fierce debate. This was highlighted early on when someone stood up in a national meeting following a presentation on the validation of the Canine Brief Pain Inventory (CBPI) and asked, “so which is better, your instrument or gait analysis?” The tone of the question and ensuing debate in the room, surprisingly captured how the proposal of a new tool, like the CBPI, could be perceived as a threat to the utility of another well validated and highly useful tool such as gait analysis which had been used in the chronic pain space for quite a long time. While we continue to get caught up in lively conversations about “subjective” vs. “objective” outcome assessment, for the most part, the scientific community now understands that it is not a question of one being better than the other. We understand that the different tools measure different things, even within a single dimension or domain (e.g., movement) that is impacted by pain. Measurement of ground reaction forces through gait analysis is the “gold standard” for measuring lameness (decreased and/or abnormal limb use), so if measuring lameness is a desirable study outcome, then gait analysis would be the optimal choice (assuming feasibility with equipment and technical expertise etc.). If a study goal is to understand how pain is impacting the ability of the animal to function in its home environment, then an owner's assessment of pain and impact on daily functions is an obvious choice. They both have pros and cons. The choice of instrument will be based on the goals of a study, the population of animals with osteoarthritis that are included, and the resources that are available to perform the assessments.

The “which is better…?” conversation around gait analysis vs. owner assessments can easily be reframed to a “what measurement or outcome makes sense for the study?” Since the development of the CBPI, other CMIs have been developed, for example, the Liverpool Osteoarthritis in Dogs (LOAD) instrument. Having multiple CMIs available comes with a new set of questions. For example, “Which is better the LOAD or the CBPI?”. While these are both owner-completed questionnaires (instruments) validated for use in dogs with osteoarthritis, the CBPI and LOAD measure different things. The CBPI asks about pain severity and impact on daily activities, while the LOAD asks about mobility in general and with exercise. They ask about different behaviors and different perspectives on those behaviors. While correlated, they are likely to work differently in populations that differ in measured and unmeasured underlying characteristics. A choice may come down to pilot data in the specific target population under study to understand which may work best.

The concept of the high level “which is better?” question is tied to the idea that there is one perfect tool to use in every situation. However, chronic pain is a multidimensional, complex condition that varies based on the underlying disease and the population being evaluated, and we ultimately need multiple valid and reliable tools to move the profession forward in the measurement and management of chronic pain for our patients (1).

Biennially, the Pain in Animals Workshop (PAW) forum brings together scientists and clinicians to focus, across veterinary species and humans, on our shared passion of improving health through our ability to recognize and monitor pain. This collaboration has been instrumental in sharing current knowledge, recognizing gaps, and aligning on the best paths forward in this challenging space.

## The pain in animals workshops (PAWs)

All PAWs have the same two foundational objectives: to advance veterinary medicine, and translational research. The veterinary medicine perspective is driven by the realization that pain, and persistent or chronic pain in particular, is one of the most poorly understood and relatively underdiagnosed conditions in animals. The translational research perspective is driven by the awareness that there is currently a lack of translation of basic research into new and approved therapeutics for the treatment of pain in humans. The workshops have taken both broad and deep dive approaches to meeting their objectives.

### 2017 PAW

The first workshop was held in 2017 and focused on the status of chronic pain measurement in companion animals from the perspectives of therapeutic drug development & translational research. It touched on the predominant methods of outcome assessment including clinical metrology instruments (CMIs), gait analysis, and activity monitoring, as well as the potentially valuable approaches of quantitative sensory testing (QST) and measuring nociceptive withdrawal reflexes. The impact of placebo effects on pain outcome measures was also addressed. The workshop focused on a broad list of topics (1) from which an extensive list of priorities for future research was generated (2).

The priorities identified in the first workshop around CMIs were categorized as (1) additional reliability and validity testing of currently available CMIs, (2) Standard Operating Procedure (SOP) development for the appropriate use of CMIs, and (3) optimal study designs for the use of CMIs. In the seven years since the workshop, there has been a lot of activity in the form of publications related to the first priority, including refining CMIs (3), measuring pain in non-musculoskeletal conditions (most commonly cancer pain or neuropathic pain), CMI use related to targeted administration of analgesics (primarily intraarticular) (4, 5), as well as efforts to understand clinically important differences and success/failure criteria when using CMIs (6, 7). Comparatively less work has been accomplished for the second and third priorities. There are no aligned, widely available guidelines for how CMIs should be used, nor are there any easily identifiable and accessible handbooks with instructions on the use of most of the currently available instruments. There are a handful of publications associated with understanding the optimal study design for the use of CMIs (8), such as response bias or recall bias (9), but clearly more needs to be done to understand optimal clinical designs for CMIs in veterinary medicine.

The priorities for activity monitoring included the need to develop metrics around what clinically relevant improvement constitutes, as well as factors to consider in the use of monitors for our veterinary species. Following the workshop, there were reports published that improved our understanding of how to use currently available activity monitors (10–16). These reports included how best to capture changes in the severity of pain, the best output to use, and the presence of signatures related to specific pain behaviors, such as night-time activity and how well animals sleep. In addition, the 2021 PAW leveraged this new information and did a deep dive into accelerometry focusing on SOPs for use and best approaches to the statistical evaluation of the data.

The priorities identified for gait analysis at the 2017 workshop focused on refining currently available techniques and how to ensure investigators are getting the best data, as well as understanding the differences in the various systems used to collect gait analysis data, and a focus on which parameters may be most appropriate as outcomes. Along with accelerometry, the 2021 PAW included a detailed discussion about gait analysis to continue to drive the conversation forward four years after that initial workshop, and this resulted in published “best practices” (17).

In 2017, the priorities for QST focused on the need to understand sensory phenotyping (2). There has not been significant activity in this area in veterinary medicine, relative to the other assessment tools discussed at the 2017 workshop, likely due to the variability of data related to the testing paradigm. Suggested protocols with video explanations have been published to standardize protocols. However, in terms of applicability of QST, there remains a fundamental need to understand whether direct treatment-related changes in sensory processing can be documented, or whether any parameters can be used to define a phenotype that predicts response to analgesics.

Finally, in 2017 there were priorities identified for understanding placebo effects. One priority focused on the opportunity to pull data from the multitude of placebo-controlled trials occurring in veterinary medicine and explore which patient and study design factors impact the placebo response. The second priority raised questions around which elements of a study that have not been a focus in veterinary medicine may be impacting the placebo response (e.g., consent form wording, study site characteristics). There is no clear evidence to support significant movement for these priorities despite placebo effect topics being addressed again in the 2019 PAW.

### 2019 PAW

The second workshop in 2019 focused on acute pain and broadened the discussion to include farm animal species. It also discussed the advances and roadblocks to the measurement of acute pain, and the potential for spontaneous conditions in animals to contribute to translational acute pain research for human therapeutics. Both subjective and objective measures were discussed including questionnaires, facial expression scales, activity monitoring, kinetic limb evaluation, complex behavioral tests, and physiological measures across species.

There were areas of robust overlap identified in the acute pain assessment measures across the species. For example, it was highlighted that there were many species for which facial expression scales were being developed, much like kinetic limb evaluation. These are areas that clearly continue to be very active across species including humans, so it will be interesting to see how this research develops moving forward, now that we are just approaching five years since that workshop. In particular, leveraging artificial intelligence and machine learning to assist in the application of some measures of the impact of acute pain was touched on briefly in 2019. This is an area that is rapidly developing and will be one focus of the 2025 meeting (http://www.PAW2025.com).

### 2021 PAW

The 2021 workshop pivoted to an in-depth discussion on a selective number of topics including accelerometry and gait analysis. As noted above, these are areas of robust activity as seen in ongoing scans of the literature, as well as what is being driven directly by these workshops. The one new topic broached in the 2021 workshop was the concept of biomarkers for pain. The ability to document the presence and severity of pain with one simple objective test is the Holy Grail of pain measurement. The human pain field has been looking at this for many years without success. However, there is now a lot of focus on generating and assessing algorithms to contemporaneously evaluate a variety of potential biomarkers to identify a composite measure that may be able to identify pain, or subjects that are at risk for chronic pain. This is an area that clearly has an enormous amount of activity in the literature and a topic that future PAW meetings will focus on.

## Translational research

**Figure 1:**
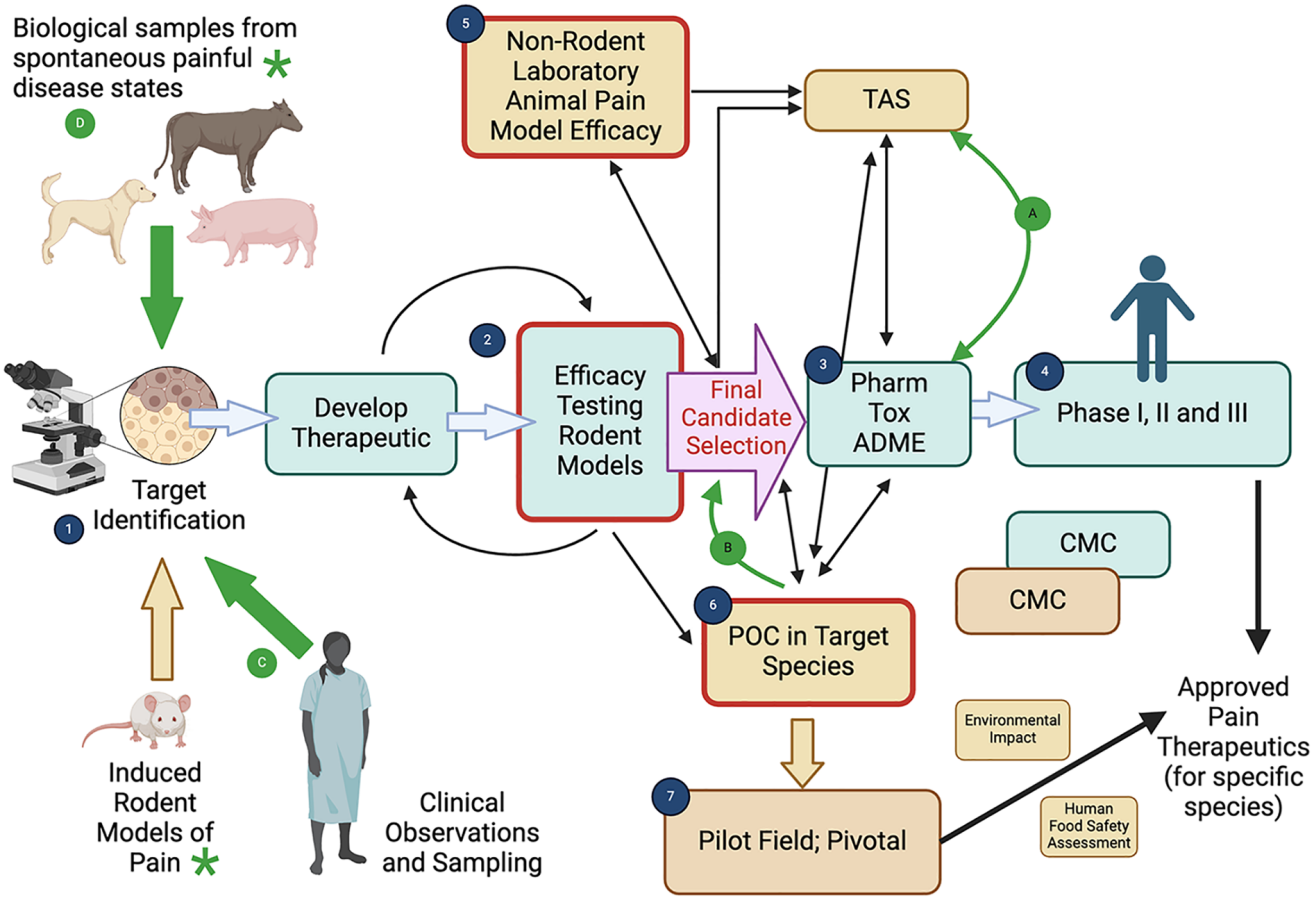
The complementary nature of human and animal pain therapeutic development paradigms. Created with BioRender.com. *Note: While sequential processes have been illustrated, the components of the therapeutic development plan often run in parallel, and the sequence of work is very varied, with results from one component informing another component. The figure is illustrative of the processes involved and designed to show the potential interactions between human and animal pain therapeutic development. Regulatory requirements may differ geographically, and between the type of drug (e.g. small molecule, biologic). The basic human pain therapeutic development paradigm (green fill boxes) consists of (1) target identification and (2) efficacy testing in rodent pain models which allows for selection of putative therapeutic(s) and further optimization may occur to finally select the lead candidate therapeutic [final candidate selection, pink fill arrow]. Human clinical testing proceeds from pharmacological, toxicological and absorption, distribution, metabolism, and excretion (ADME) characterization (3) to clinical research in Phases I, II and III (4) Chemistry, Manufacturing and Controls (CMC) is a critical component of therapeutic development and the approval process. Veterinary pain therapeutic development (brown fill boxes) follows an initially similar path, with information from induced rodent models (1,D) being used to identify targets and initiate therapeutic development (2). Following initial efficacy testing in rodent models induced pain model proof of concept studies may be used in laboratory animals (5) to determine whether the therapeutic has efficacy in the target species, provide preliminary safety information, and select a dosage regimen for the candidate therapeutic. Using the final formulation and knowledge about the target dose, target animal saftey (TAS) studies and effectiveness studies are performed in the target species (6) (which can sometimes be induced models). The effectiveness studies in the target species (6) may be performed in pilot field studies. The final effectiveness studies are adequate and well-controlled studies, which are typically field efficacy studies in the target population under the proposed conditions of use, (7), leading to potential approval. As for human drugs, CMC is critical for approval and the impact on the environment is assessed. For food-producing animals, a human food safety assessment is also required. These two development paths, while separate, can clearly be complementary. Nonclinical laboratory pharmacology and toxicology studies conducted in support of human product development (3) (which often include dogs) may be useful to provide target animal safety information (for the same species, e.g. dogs) and may also be useful as part of pharmacology/toxicology characterization in the development of a target animal safety profile (A). Conversely, nonclinical laboratory studies conducted for veterinary product development (target animal safety studies) (A) and preliminary effectiveness data in the target animal species (B) (5, 6), including pharmacology/toxicology information may help direct protocol design, indication selection, candidate drug development and dosage determinations for human pain therapeutic development. In particular, proof of concept (POC) and dosage determination studies in naturally occurring painful disease states in animals (ones that have high similarity with the human condition) can help inform appropriate candidate selection for these human products (B) (19). The PAW meetings have focused on the measurement of pain (measurement of the impact of pain) in animals, both in rodent models and non-rodent animals. *As can be seen from the position of the boxes outlined in red in the figure (2, 5, 6), the ability to appropriately measure the impact of pain (and the efficacy of analgesic therapeutics) in animals is absolutely critical to the appropriate selection of a candidate analgesic, and to the success of human clinical development programs, as well as the development of therapeutics for animals.* The figure also illustrates that the whole development program for both humans and animals depends on identification of a relevant target. While this has historically relied upon information garnered from induced rodent models of pain, neurobiological insights from human tissues and fluids can help to identify an appropriate target (C). Additionally, a hitherto untapped resource is the identification of relevant targets from tissues and fluids in naturally occurring pain states in non-rodent animals (D). *Critical to the effective utilization of tissues from any animal model or species for identifying targets is the ability to accurately measure pain in a clinically relevant manner (green asterix).*

Pain conditions in humans, particularly persistent/chronic pain are a major public health concern, with significant individual, societal and economic impacts. For persistent/chronic pain in particular, the current practice of translational biomedical research is not producing novel therapeutics, despite the medical need. Indeed, it has recently been stated that “despite generating a plethora of novel analgesic targets, pharmaceuticals for chronic pain treatment remain largely limited to the same 6 drug classes as present 40 years ago” (18). The failure of translational pain research has been discussed with emphasis on the models and outcome measures in pre-clinical research, with repeated calls for improved, validated, clinically relevant methods to measure pain. The majority of preclinical models are induced (i.e., created) to model or mimic the target clinical conditions. While these have worked well for increasing mechanistic understanding of disease, they do not appear to have worked as well for selecting new analgesic drug candidates. This has led to suggestions of utilizing spontaneous disease models in non-rodent animals to help inform pain therapeutic development (19–21). If these non-rodent models are to be useful, we need reliable, valid, and clinically relevant methods to measure pain. Therefore, for the aforementioned reasons, the focus at PAW forums on discussions to advance the measurement of pain in animals is highly relevant to translational research. Further, one of the clear outcomes of the PAW meetings has been greater transdisciplinary appreciation and understanding of the complementary nature of human and animal pain therapeutic development paradigms and the opportunities for enriching both through closer collaboration (Figure 1).

## Discussion

Looking at the timeline of pain assessment and management for veterinary species, quite a lot has been accomplished in the veterinary community in just 25 years, including increasing awareness of the importance of veterinary medicine to translational research and human health. There is obviously still much to learn, particularly through collaboration, data sharing, and open communication. The Pain in Animals Workshops have been integral to the most recent wave of knowledge gain and awareness. The biennial organization of the PAWs around shared learnings and robust discussions across species drives progression and innovation in the space that is not possible in inwardly focused and siloed research programs alone. The passionate commitment to robust cross-species scientific sharing can drive improved health for all our patients.

## Data Availability

The original contributions presented in the study are included in the article/Supplementary Material, further inquiries can be directed to the corresponding author.
